# A Low Power Energy-Efficient Precision CMOS Temperature Sensor †

**DOI:** 10.3390/mi9060257

**Published:** 2018-05-24

**Authors:** Rongshan Wei, Xiaotian Bao

**Affiliations:** College of Physics and Information Engineering, Fuzhou University, Fuzhou 350116, Fujian, China; n161120014@fzu.edu.cn

**Keywords:** mode matching methods, temperature sensor, calibration

## Abstract

This paper presents a low power, energy-efficient precision CMOS temperature sensor. The front-end circuit is based on bipolar junction transistors, and employs a pre-bias circuit and bipolar core. To reduce measurement errors arising from current ratio mismatch, a new dynamic element-matching mode is proposed, which dynamically matches all current sources in the front-end circuit. The first-order fitting and third-order fitting are used to calibrate the output results. On the basis of simulation results, the sensor achieves 3σ-inaccuracies of +0.18/−0.13 °C from −55 °C to +125 °C. Measurement results demonstrate sensor 3σ-inaccuracies of ±0.2 °C from 0 °C to +100 °C. The circuit is implemented in 0.18 μm CMOS, and consumes 6.1 μA with a 1.8 V supply voltage.

## 1. Introduction

Knowing the die temperature of transistors employed in precision sensor systems is often quite important because this information can be used to mitigate the cross sensitivity of a system to temperature [1,2]. In this manner, temperature sensors have been employed to compensate for the temperature dependence of MEMS resonators [1], to compensate for the curvature in a band-gap voltage reference [2], or in temperature measurements and over-temperature protection directly. In such systems, the inaccuracy of temperature sensors is a significant component of the total error budget, and thus often limits their ultimate performance.

Wu et al. [3] presented a thermistor-based sensor that achieved an inaccuracy of ±0.5 °C from 0 °C to 100 °C with one-point calibration. Although a thermistor-based sensor has the advantage of low power consumption, it usually requires multi-point calibration to attain high accuracy because of the non-linearity between resistance and temperature. Chen et al. [4] proposed a time-to-digital converter based temperature sensor that achieved an inaccuracy of −0.7/+0.9 °C from 0 °C to 100 °C. Testi et al. [5] presented a ring oscillator-based temperature sensor that achieved a maximum inaccuracy of ±3 °C from 0 °C to 120 °C after two-point calibration. While both of these latter temperature sensors have the advantage of low power consumption, they failed to achieve high accuracies.

Sensors based on bipolar junction transistors (BJTs) can achieve high accuracy because the base-emitter voltage *V_BE_* offers advantageous temperature characteristics. Lee et al. [6] proposed a BJT-based temperature sensor that achieved an inaccuracy of ±1 °C from −55 °C to 125 °C. Aita et al. [7] presented a BJT-based temperature sensor that achieved an inaccuracy of ±0.25 °C from −70 °C to 130 °C using dynamic element matching (DEM). 

This paper proposes a temperature sensor circuit based on BJTs for operation over the temperature range of 0 °C to 100 °C. Compared with the DEM approaches employed previously to reduce mismatch related errors [8,9], we propose a new DEM approach, denoted as all DEM (ALL-DEM), that provides greater temperature measurement precision by dynamically matching all current sources in the front-end circuit. Moreover, compared with the employment of a second-order sigma-delta analog-to-digital converter (ADC) [10] and a first-order zoom ADC [11], the sensor employs a second-order zoom ADC, which can improve the resolution and reduce the required conversion time. In addition, the temperature sensor employs voltage calibration to improve its performance.

The remainder of this paper is organized as follows. Section 2 describes the operation of the BJT-based temperature sensor front-end circuit, and discusses its main error sources. Section 3 discusses the proposed temperature sensor front-end circuit employing the ALL-DEM approach in detail. Section 4 describes the structure and operating modes of a zoom ADC. Section 5 discusses the voltage calibration and the means of conducting first-order fitting and third-order fitting. Section 6 presents the simulation and measurement results of the chip and discusses this work. Section 7 concludes the paper.

## 2. Sensor Operating Principles and Error Budgeting 

**Figure 1:**
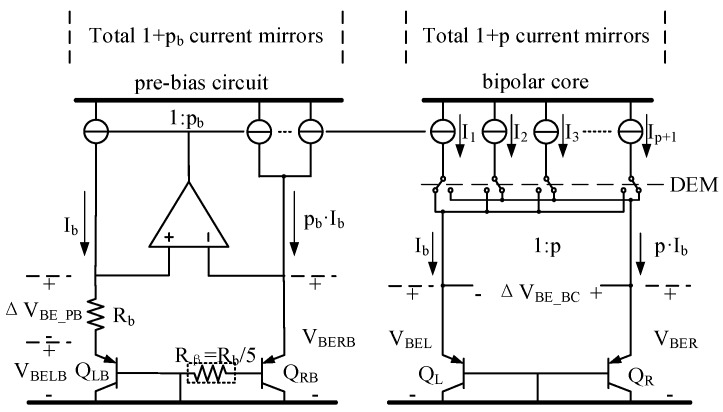
Conventional bipolar junction transistor (BJT) based temperature sensor front-end circuit.

As shown in Figure 1, the front-end circuit consists of a pre-bias circuit and a bipolar core built around several current sources, four substrate PNP transistors, and an opamp. Two branches of current with a ratio of 1:*p_b_* bias a pair of PNPs (Q_LB_ and Q_RB_). 

The base-emitter voltages *V_BE_* of the PNPs (*V_BELB_* and *V_BERB_*) are complementary to the absolute temperature (CTAT), whereas the difference between the two values of *V_BE_*, denoted as ∆*V_BE_PB_*, is proportional to the absolute temperature (PTAT). For a BJT, these voltages can be given by

(1) $$V_{BE}=\eta \frac{kT}{q}ln\left(\frac{I_{C}}{I_{S}}\right)$$

(2) $$\Delta V_{BE\_PB}=V_{BERB}-V_{BELB}=\eta \frac{kT}{q}ln\left(p_{b}\right)$$

respectively, where *η* is a process-dependent nonideality factor, *k* is the Boltzmann constant, *q* is the electron charge, *T* is the temperature in Kelvin, and *I_C_* and *I_S_* are the collector and saturation currents of the BJT. An opamp forces ∆*V_BE_PB_* across a resistance of *R_b_* to generate a PTAT bias current *I_b_* = ∆*V_BE_PB_*/*R_b_*. The PTAT current is mirrored to the bipolar core, and two current branches with a ratio of 1:*p* are directed to two PNPs (Q_L_ and Q_R_) to generate two additional *V_BE_* (*V_BEL_* and *V_BER_*) and ∆*V_BE_BC_* values.

Because the current gain *β* of the PNPs is finite and the biasing current is directed to a PNP via its emitter, the ratio of *I_C_* (Q_LB_ and Q_RB_) is not the same as a current branch. Using a *β*-compensating resistance of *R_β_* = *R_b_*/5 in the pre-bias circuit suppresses the effect of *β* on *V_BE_* [12].

**Figure 2:**
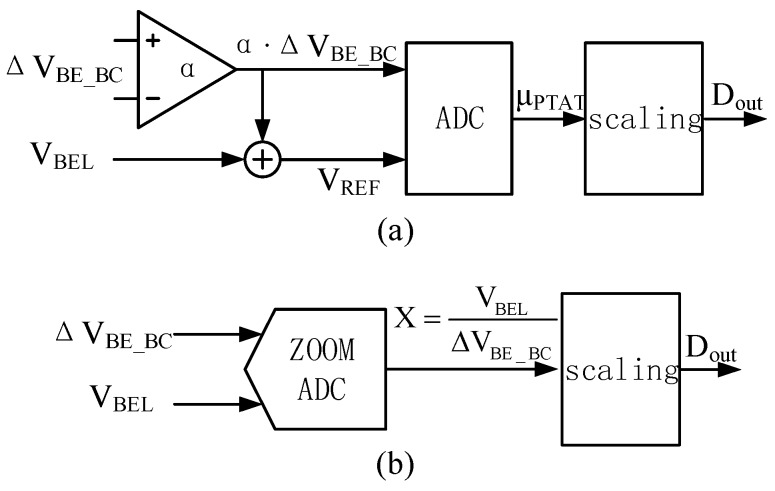
Operating principles of analog-to-digital converters (ADCs): (**a**) sigma-delta ADC; (**b**) zoom ADC.

Generally, as shown in Figure 2a, temperature can be measured as α·∆*V_BE_* with respect to a reference voltage *V_REF_* = *V_BEL_* + α·∆*V_BE_BC_*, where α is a constant [9]. This provides the parameter *μ_PTAT_* = α·∆*V_BE_BC_*/*V_REF_*, which varies linearly from ~0.3 to ~0.7 over the temperature range considered [12]. Alternatively, as shown in Figure 2b, a zoom ADC is employed for this purpose to reduce the power consumption, die area, and required conversion time [8,13]. 

The ADC provides the parameter *X* = *V_BEL_*/∆*V_BE_BC_*, which varies nonlinearly from ~28 to ~8 over the temperature range considered [8]. In this case, the parameter *μ_PTAT_* can be determined in the digital back-end according to the relationship *μ_PTAT_* = *X*/(*α* + *X*), where *α* is a calibration parameter. The discretized output *D_out_* based on the parameter *μ_PTAT_* can then be converted to units of °C by a linear fit as follows [8]:

(3) $$D_{out}=A\cdot \mu_{PTAT}+B=A\cdot \frac{\alpha }{X+\alpha }+B=A\cdot \frac{\alpha \cdot \Delta V_{BE\_BC}}{V_{BEL}+\alpha \cdot \Delta V_{BE\_BC}}+B$$

Here, *A* and *B* are calibration parameters with approximate values of 600 and −273, respectively.

According to Equation (3), the sensitivity of *D_out_* to error in *V_BEL_* and ∆*V_BE_BC_* can be respectively expressed by

(4) $$S_{V_{BEL}}^{D_{out}}\left(T\right)=\frac{\partial D_{out}}{\partial V_{BEL}}=A\cdot \frac{\partial \mu_{PTAT}}{\partial V_{BEL}}=-\frac{T}{V_{REF}}$$

(5) $$S_{\Delta V_{BE\_BC}}^{D_{out}}\left(T\right)=\frac{\partial D_{out}}{\partial \Delta V_{BE\_BC}}=A\cdot \frac{\partial \mu_{PTAT}}{\partial \Delta V_{BE\_BC}}=\frac{A-T}{V_{REF}}\alpha$$

where the approximation *μ_PTAT_* ≈ *T*/*A* has been employed in the final forms. For example, supposing that *V_REF_* = 1.2 V, *A* = 600, and *α* = 14, a 0.1 °C temperature error is approximately equal to a 0.3 mV error in *V_BEL_* at +126.85 °C or a 0.02 mV error in ∆*V_BE_BC_* at −73.15 °C. Therefore, the accuracy of the sensor is limited by the error in *V_BEL_* and ∆*V_BE_BC_*.

The main source of error in ∆*V_BE_BC_* is a current ratio mismatch ∆*p* between the two current branches in the bipolar core. The absolute error in ∆*V_BE_BC_* can then be given as follows:

(6) $$\Delta V_{BE\_BC}-\Delta V_{BE\_BC}{|}_{\Delta p=0}=\eta \frac{kT}{q}ln\left(p+\Delta p\right)-\eta \frac{kT}{q}ln\left(p\right)=\eta \frac{kT}{q}ln\left(1+\frac{\Delta p}{p}\right)\approx \eta \frac{kT}{q}\frac{\Delta p}{p}$$

Here, *T* is in units of Kelvin. With a carefully designed layout of the current sources, ∆*p*/*p* = 0.1% can be expected [14], and the temperature error due to mismatch is then 0.091 K according to Equation (5).

Error due to ∆*p* can be reduced by dynamically interchanging the current sources in the bipolar core [15] using DEM [16]. This is illustrated for the case of the bipolar core in Figure 1. One of the current sources is directed to Q_L_, whereas the other current sources are directed to Q_R_. This averaging process cancels the first-order error in ∆*V_BE_BC_* whereas the second-order error remains, which is given as follows [12]:

(7) $$\left|\Delta V_{BE\_BC}-\Delta V_{BE\_BC}{|}_{\Delta p=0}\right|<\frac{1}{2}\eta \frac{kT}{q}{\left(\frac{\Delta p}{p}\right)}^{2}$$

If, for instance, ∆*p*/*p* = 1%, this corresponds to a temperature error of at most 4.6 mK, which is sufficient to obtain a temperature error well below 0.1 K.

Similarly, a current ratio mismatch exists in the pre-bias circuit, which results in PTAT errors in *I_b_* and *V_BEL_*. The absolute error in *V_BEL_* can then be given as follows:

(8) $$V_{BEL}-V_{BEL,ideal}=\eta \frac{kT}{q}ln\left(\frac{p+\Delta p}{p}\right)\approx \eta \frac{kT}{q}\cdot \frac{\Delta p}{p}$$

If, for example, ∆*p*/*p* = 1%, the error in *V_BEL_* corresponds to a temperature error of at most 65 mK.

To reduce temperature measurement errors, most conventional circuits match only current sources in the bipolar core dynamically [8,9,13], and the mismatch in the pre-bias circuit is regarded as a relatively minor problem.

## 3. Temperature Sensor Front-End Circuit

**Figure 3:**
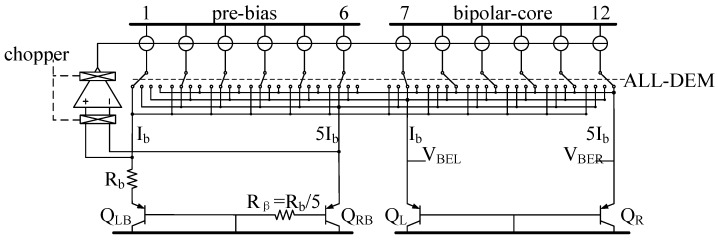
Front-end circuit employing the all dynamic element matching (ALL-DEM) approach.

According to the discussion in the previous section, a conventional circuit reduces temperature measurement errors by averaging ∆*p* to some extent, but cannot make full use of the circuit because current sources in the pre-bias circuit do not participate in the averaging process. Therefore, we employed the proposed ALL-DEM approach in the temperature sensor front-end circuit, which matches all current sources in both the pre-bias circuit and bipolar core dynamically. Assuming that *p_b_* = *p* = 5, the proposed circuit is illustrated in Figure 3.

The ALL-DEM approach employs the following steps:

Step 1: Number all current sources from 1 to 12.

Step 2: Under the control of ALL-DEM, current source 1 is directed to Q_LB_, current sources 2–6 are directed to Q_RB_, current source 7 is directed to Q_L_, and current sources 8–12 are directed to Q_R_.

Step 3: After a single integral sampling period, current sources are cyclically shifted, and Step 2 is repeated, i.e., current source 2 is directed to Q_LB_, current sources 3–7 are directed to Q_RB_, current source 8 is directed to Q_L_, and current sources 9–12 and current source 1 are directed to Q_R_.

An ALL-DEM cycle consists of 12 periods. In addition to dynamically matching the current sources in the pre-bias circuit (rather than only in the bipolar core), the ALL-DEM approach offers another advantage, in that it provides a greater number of conditions in the bipolar core compared with conventional DEM, which is increased from 6 to 12. 

**Figure 4:**
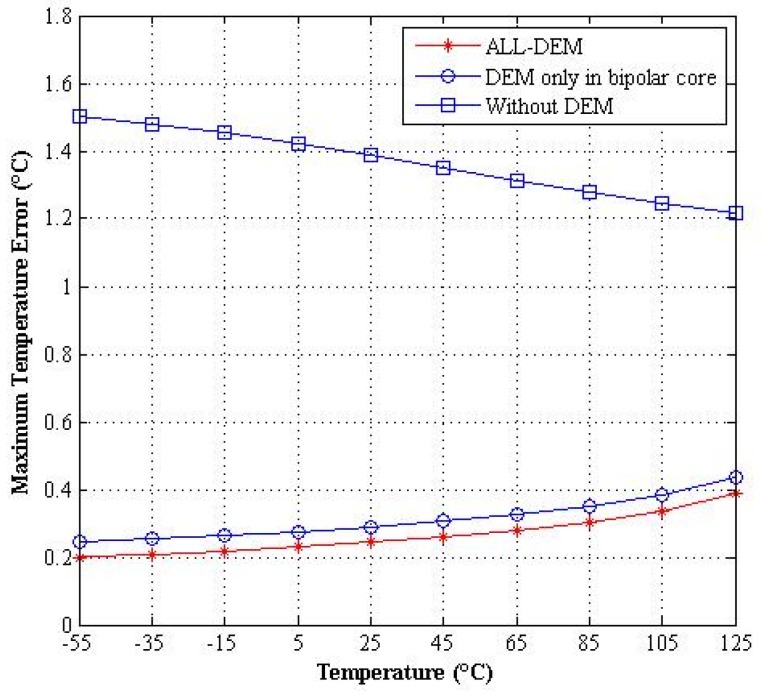
Maximum temperature errors simulations with different dynamic element matching (DEM) schemes.

The ALL-DEM approach can reduce the mismatch in the pre-bias circuit, and, hence, can reduce temperature measurement errors caused by the pre-bias circuit. In addition, the proposed circuit employs chopping to suppress the offset of the opamp. The benefits of the ALL-DEM approach are demonstrated by the simulation results presented in Figure 4, which shows the maximum temperature errors obtained with different DEM schemes. As the results show, the maximum temperature error caused by the ALL-DEM approach is at least 0.82 °C less than that obtained without the DEM approach. Moreover, the average maximum temperature error caused by the ALL-DEM approach is 0.07 °C less than that of the conventional DEM approach. The ALL-DEM approach can therefore achieve a higher accuracy than the conventional DEM approach.

## 4. Zoom Analog-to-Digital Converter

**Figure 5:**
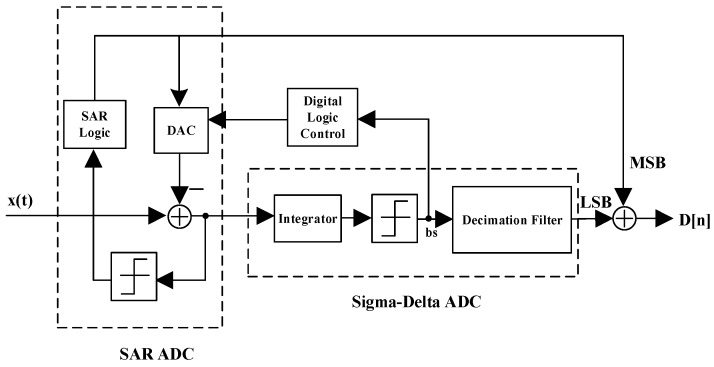
Structure of the zoom ADC. (Note: SAR: successive approximation; LSB: least significant bit; MSB: most significant bit; DAC: digital-to-analog converter).

As shown in Figure 5, the zoom ADC is a two-step ADC structure that consists of a successive approximation (SAR) ADC and a sigma-delta ADC. The conversion process of the zoom ADC can be divided into two operating modes. Here, the SAR ADC first converts the input *x*(*t*) to obtain the output of the most significant bit (*MSB*), which is denoted as coarse conversion. Then, the zoom ADC enters into the second operating mode, where the sigma-delta ADC converts the input *x*(*t*) according to *MSB*, and generates the output of the least significant bit (*LSB*), which is denoted as fine conversion. Finally, we obtain the output *D*[*n*] by combining *MSB* and *LSB*. Therefore, the zoom ADC combines the rapid conversion of an SAR ADC with the high precision of a sigma-delta ADC.

## 5. Calibration

**Figure 6:**
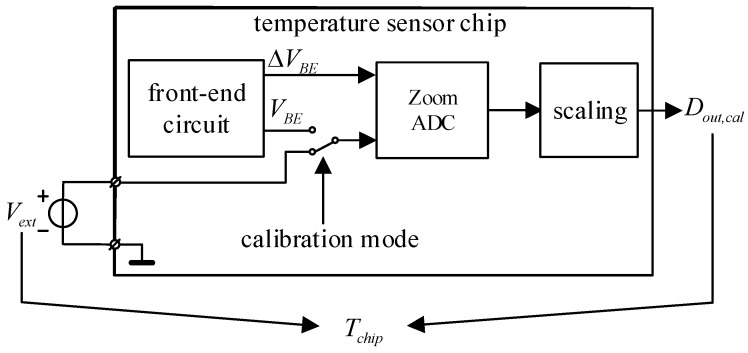
Overview of the temperature sensor.

Figure 6 presents an overview of the temperature sensor. It consists of a front-end circuit that generates *V_BE_* and ∆*V_BE_*, which are fed to the zoom ADC. The output of the zoom ADC in the normal operating mode is the ratio *X_N_* = *V_BE_*/∆*V_BE_*. However, the ADC can be configured to output *X_c_* = *V_ext_* /∆*V_BE_* when the temperature sensor chip is placed in the calibration mode. A PT-100 thermistor, which was calibrated to an error of less than 1 mK and placed in good thermal contact with the temperature sensor, was used to obtain the reference temperature *T_chip_* [17].

We adopted voltage calibration, which employs the following steps.

Step 1: Change the operating mode of the temperature sensor chip into the calibration mode. Then, input testing voltage *V_ext_*, and obtain ∆*V_BE_* as follows:

(9) $$\Delta V_{BE}=\frac{V_{ext}}{X_{ext}}$$

We can then calculate *η* using ∆*V_BE_* and *T_chip_* according to Equation (2).

Step 2: Change the operating mode of the temperature sensor chip into the normal operating mode. Take 20 temperature reading samples over 11 testing points in the temperature range from 0 °C to +100 °C, and obtain *X* and *D_out_* from each testing point, respectively. The average value *X_AVG_* at each testing point can be calculated from the values of *X* obtained at each testing point. Then, the calibration parameters *A*, *B*, and *α* can be calculated according to Equation (3).

Step 3: The voltage calibration temperature *T_D_* can be calculated using *η*, Equations (2) and (9).

Step 4: Then, the ideal voltage calibration output *X_ideal_* can be calculated using *T_D_*, *A*, *B*, and *α* according to Equation (3). In addition, we can obtain the actual output *X_D_* from the chip.

Step 5: Output *D* is then obtained from *A*, *B*, *α*, *X_ideal_*, and *X_D_* as follows:

(10) $$D=A\cdot \frac{\alpha }{X+X_{ideal}-X_{D}+\alpha }+B$$

Here, Equation (10) represents the first-order fitting. Actually, the fitting accuracy can be improved by adopting the following fixed third-order polynomial:

(11) $$D=A\cdot {\left(\frac{\alpha }{X^{'}+\alpha }\right)}^{3}+B\cdot {\left(\frac{\alpha }{X^{'}+\alpha }\right)}^{2}+C\cdot \left(\frac{\alpha }{X^{'}+\alpha }\right)+E$$

where *X^’^* = *X* + *X_ideal_* − *X_D_*, *A*, *B*, *C*, and *E* are calibration parameters that are calculated in Step 1. In this paper, the voltage calibration employed single-point calibration, and the value *T_D_* was set to 37 °C.

## 6. Experimental Results and Discussion

**Figure 7:**
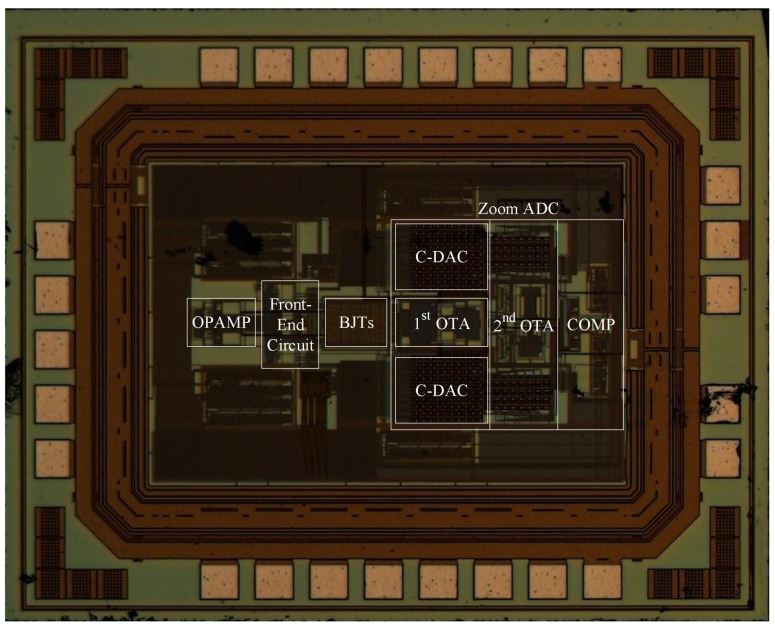
Chip micrograph. (Note: OTA: operational transconductance amplifier; C-DAC: capacitive digital-to-analog converter; COMP: comparator).

The circuit was implemented in 0.18 μm CMOS technology provided by Semiconductor Manufacturing International Corp. (SMIC, Shanghai, China), and all current sources employed a current of 180 nA. The chip consumed 6.1 μA with a 1.8 V supply voltage, where the current consumption of the front-end circuit was 4.6 μA and the current consumption of the zoom ADC was 1.5 μA. The core area of the chip was 860 μm × 580 μm. A micrograph of the chip is shown in Figure 7.

**Figure 8:**
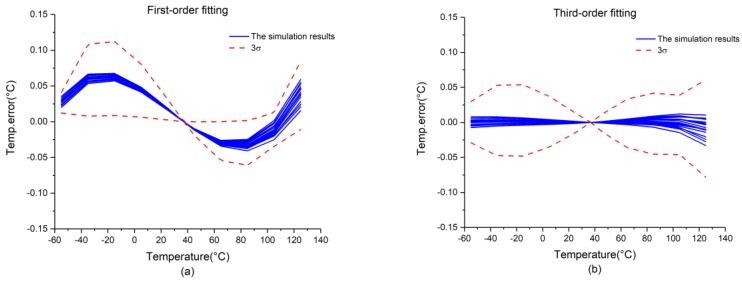
Simulation results of the front-end circuit: (**a**) first-order fitting; (**b**) third-order fitting.

Figure 8 presents the simulation results of the front-end circuit obtained via 20 Monte Carlo simulations. Here, Figure 8a presents the simulation results of the first-order fitting, which exhibits 3σ-inaccuracies of +0.12/−0.06 °C from −55 °C to +125 °C. In addition, Figure 8b presents the simulation results of the third-order fitting, which exhibits 3σ-inaccuracies of ±0.08 °C from −55 °C to +125 °C.

**Figure 9:**
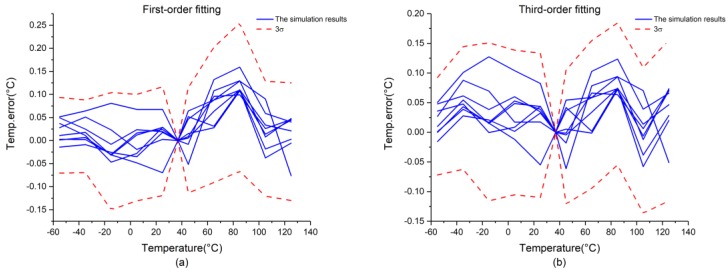
Simulation results for the chip: (**a**) first-order fitting; (**b**) third-order fitting.

The simulation results of the chip are presented in Figure 9. Here, Figure 9a presents the results of first-order fitting, which exhibits 3σ-inaccuracies of +0.25/−0.13 °C over the temperature range considered. In addition, Figure 9b presents the results of third-order fitting, which exhibits 3σ-inaccuracies of +0.18/−0.13 °C over the temperature range considered.

**Figure 10:**
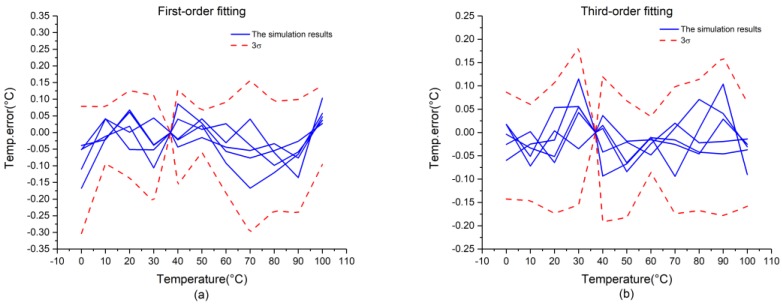
Measurement results of the chip: (**a**) first-order fitting; (**b**) third-order fitting.

Figure 10 presents the measurement results of the chip. During measurements, we added a shielding box to improve the accuracy of the temperature sensor. As shown in Figure 10a, the chip exhibits 3σ-inaccuracies of +0.15/−0.3 °C from 0 °C to +100 °C with first-order fitting. Figure 10b presents the measurement results of third-order fitting, which shows that the chip can achieve 3σ-inaccuracies of ±0.2 °C from 0 °C to +100 °C.

As demonstrated by the simulation and measurement results, third-order fitting can achieve a greater measurement than first-order fitting. The measured performance of the temperature sensor is compared with the performances of previously reported state-of-the-art temperature sensor implementations in Table 1. Because of the employment of the second-order zoom ADC, the parameter figure-of-merit (FOM) of the sensor is better than those presented in References [6,15], which shows that the proposed sensor provides better resolution and a reduced conversion time. However, the measurement temperature range of this work is only from 0 °C to 100 °C because of the limitations of the measurement equipment. As a result, the *Rel.InAcc* value of the proposed circuit is 0.4%.

The present work has taken errors into account as much as possible and employs the ALL-DEM approach and calibration to improve the accuracy of the proposed temperature sensor. Among the remaining errors, the curvature error is the largest source of error. Therefore, a curvature calibration or a curvature compensation technology can be expected to improve the accuracies of the temperature sensor. In addition, while the BJT-based temperature sensors provide higher accuracy than thermistor-based sensors, they also require greater power consumption. A higher-order zoom ADC can reduce the power consumption, although its implementation will also require a greater die area. The miniaturization of BJT-based temperature sensors is also an important consideration, and the means of reducing the die area while simultaneously reducing the power consumption of the temperature sensor will be the objective of future research.

## 7. Conclusions

This paper proposed a low power, energy-efficient precision CMOS temperature sensor circuit implemented in 0.18 μm CMOS technology provided by SMIC. Using the novel ALL-DEM approach, all current sources in the proposed front-end circuit are matched dynamically to reduce system errors arising from current ratio mismatch. Single-point calibration was adopted to improve the accuracy of the sensor. According to the experimental results, the proposed circuit provides a maximum temperature error of ±0.2 °C over the temperature range from 0 °C to +100 °C. The proposed circuit is applicable to a wide range of temperature sensor applications.

## Figures and Tables

**Table 1:** Performance Comparison with Previously Reported Temperature Sensor Schemes.

Item	[8]	[9]	[17]	[18]	This Work
Year	2013	2005	2017	2014	2018
Process (μm)	0.16	0.7	0.16	0.7	0.18
Area (mm^2^)	0.08	4.5	0.16	0.8	0.5
V_DD_ (V)	1.5–2	2.5–5.5	1.5–2	2.9–5.5	1.8
Supply Current (μA)	3.4	75	4.6	55	6.1
Temperature Range (°C)	−55 to +125	−55 to +125	−55 to +125	−45 to +130	0 to +100
Resolution (°C) T_conv_ (ms)	0.02 (5.3)	0.01 (100)	0.015 (5)	0.003 (2.2)	0.01 (3.4)
Inaccuracy (°C)	±0.15	±0.1	±0.06	±0.15	±0.2
FOM * (pJ°C^2^)	11	1875	7.8	3.2	3.8
Rel.InAcc. ** (%)	0.17	0.11	0.07	0.17	0.4

* FOM = (Power·T_conv_) × (Resolution)^2^. ** Rel.InAcc = (2 × Inaccuracy/Range) × 100%.
